# Genome of the four-finger threadfin *Eleutheronema tetradactylum* (Perciforms: Polynemidae)

**DOI:** 10.1186/s12864-020-07145-1

**Published:** 2020-10-19

**Authors:** Zhe Qu, Wenyan Nong, Yifei Yu, Tobias Baril, Ho Yin Yip, Alexander Hayward, Jerome H. L. Hui

**Affiliations:** 1 School of Life Sciences, Simon F.S. Li Marine Science Laboratory, State Key Laboratory of Agrobiotechnology, The Chinese University of Hong Kong, Hong Kong, China; 2 Centre for Ecology and Conservation, University of Exeter, Penryn Campus, Penryn, Cornwall, Exeter, TR10 9FE UK

**Keywords:** *Eleutheronema tetradactylum*, Fish, Genome, Transcription factor, MicroRNA, Repetitive element

## Abstract

**Background:**

Teleost fish play important roles in aquatic ecosystems and aquaculture. Threadfins (Perciformes: Polynemidae) show a range of interesting biology, and are of considerable importance for both wild fisheries and aquaculture. Additionally, the four-finger threadfin *Eleutheronema tetradactylum* is of conservation relevance since its populations are considered to be in rapid decline and it is classified as endangered. However, no genomic resources are currently available for the threadfin family Polynemidae.

**Results:**

We sequenced and assembled the first threadfin fish genome, the four-finger threadfin *E. tetradactylum*. We provide a genome assembly for *E. tetradactylum* with high contiguity (scaffold N50 = 56.3 kb) and high BUSCO completeness at 96.5%. The assembled genome size of *E. tetradactylum* is just 610.5 Mb, making it the second smallest perciform genome assembled to date. Just 9.07–10.91% of the genome sequence was found to consist of repetitive elements (standard RepeatMasker analysis vs custom analysis), making this the lowest repeat content identified to date for any perciform fish. A total of 37,683 protein-coding genes were annotated, and we include analyses of developmental transcription factors, including the *Hox*, *ParaHox*, and *Sox* families. MicroRNA genes were also annotated and compared with other chordate lineages, elucidating the gains and losses of chordate microRNAs.

**Conclusions:**

The four-finger threadfin *E. tetradactylum* genome presented here represents the first available genome sequence for the ecologically, biologically, and commercially important clade of threadfin fish*.* Our findings provide a useful genomic resource for future research into the interesting biology and evolution of this valuable group of food fish.

**Supplementary information:**

**Supplementary information** accompanies this paper at 10.1186/s12864-020-07145-1.

## Background

Teleostei is the most species-rich and diverse group of vertebrates, with ~ 30,000 species, accounting for around half of all extant vertebrate species [1]. In addition to their great diversity, teleosts play important ecological roles in aquatic ecosystems, and are of great relevance as a source of protein in both wild fisheries and aquaculture. Consequently, the study of teleost genomes is of considerable importance from evolutionary, ecological and applied perspectives.

The teleost order Perciformes comprises more than 10,000 species and represents the single largest group of vertebrates, with representatives in almost every aquatic ecosystem on earth [2]. Many perciform fish are highly important commercially, and the group is fast becoming an important model system for vertebrate genomics, with the number of genome sequences available growing rapidly.

The fourfinger threadfin, *Eleutheronema tetradactylum*, belongs to the perciform family Polynemidae, commonly known as threadfins. A distinguishing feature of threadfins is the pectoral fin, which is composed of two distinct sections, an upper normally shaped fin, and a lower section consisting of up to seven long, threadlike independent rays, which are believed to act as sensory probes for locating food in muddy habitats [3]. Like other threadfins, *E. tetradactylum* has the ability to tolerate a wide range of salinities, and is often found in estuaries and rivers, as well as its main coastal marine habitat over shallow sand or mud flats [4, 5]. Threadfins are important for commercial and sport fisheries across a wide socio-economic spectrum, and are marketed as fresh, frozen, dried or salted fish. *E. tetradactylum* is a particularly valued species, not least because of its large size compared to other threadfins (~ 2 m maximum length). However, *E. tetradactylum* is believed to be declining rapidly across much of its tropical Indo-West Pacific range, and is classified as endangered by the IUCN [6]. Like many fish, *E. tetradactylum* is a protandrous hermaphrodite that can undergo sex change at different ages [4, 7].

To date, no genomic resources are available for the threadfin family Polynemidae. To address this and provide the first reference genome for Polynemidae, we sequenced and assembled a draft genome for the four-finger threadfin, *Eleutheronema tetradactylum*. Here we describe the *E. tetradactylum* genome, to provide resources that can facilitate a better understanding of this poorly studied fish lineage. We compare findings to those for other perciform fish, and perform a range of specific analyses on transposable element content, and key developmental loci, including Hox genes and microRNAs.

## Results

### Genome assembly metrics

The genomic DNA from a single individual of *E. tetradactylum* was isolated and sequenced using the 10X Genomics platform. The final assembly size was 610.5 Mb, which is close to the estimated genome size (~ 630 Mb, Supplementary Figure S1). The scaffold N50 length was 56.3 kb (Table 1), and among the 36,746 scaffolds, the longest scaffold was 568.4 kb. To estimate genome completeness, we performed a BUSCO assessment [8]. We found that 96.5% of BUSCO genes were detected (88.2% of which were complete, and 8.3% of which were fragmented) (Table 1, Supplementary Table S1).

**Table 1:** Summary of genome assembly metrics for *E. tetradactylum*

Common name Species	Four-finger threadfin *Eleutheronema tetradactylum*
Accession number	WFKG00000000
Number of scaffolds	36,746
Assembly size	610,497,648
Scaffold N50	56,314
Largest scaffold	568,375
Number of genes (protein-coding game)	38,490 (37,683)
Gap content	14,703
BUSCOs (Complete)	96.5% (88.2)%

**Fig. 1:**
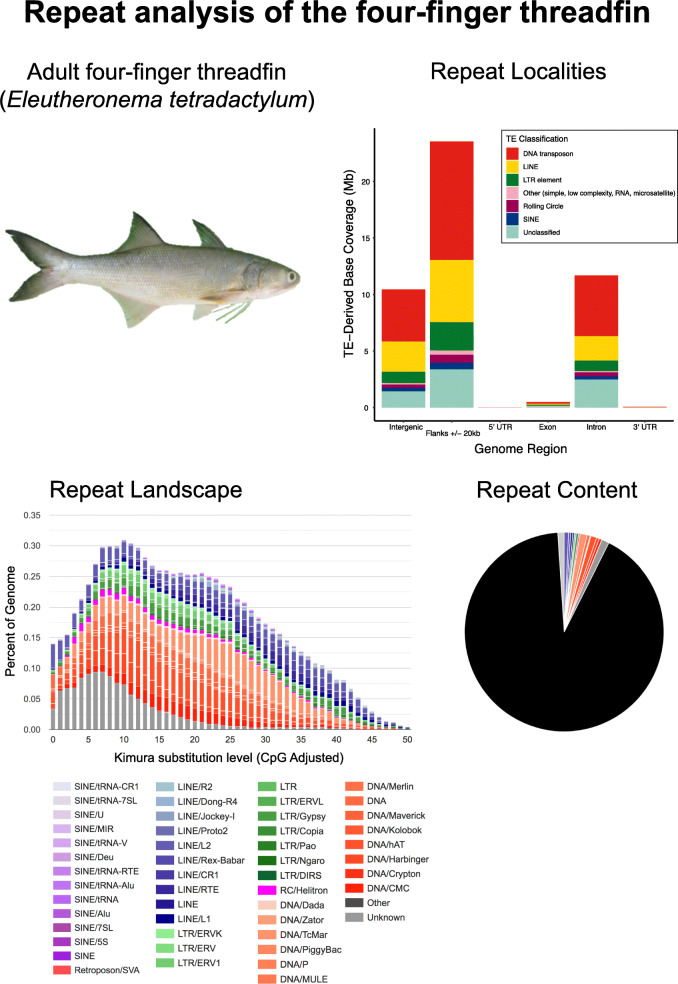
Comparison of orthologous gene groups between *E. tetradactylum* and other vertebrates. **a** Shared and unique orthologous gene groups in four species of Teleostei and human. **b** Genome-wide comparison of orthologous genes among various percid fish. Abbreviations: Cau: goldfish *Carassius auratus*; Dre: zebrafish *Danio rerio*; Ete/Etet: four-finger threadfin *Eleutheronema tetradactylum*; Hsa: human *Homo sapiens*; Ssa: Atlantic salmon *Salmo salar*; Carg: *Channa argus*; Ecra: *Etheostoma cragini*; Espe: *Etheostoma spectabile*; Lcro: *Larimichthys crocea*; Lmac: *Lateolabrax maculate*; Nalb: *Nibea albiflora*; Pcha: *Parachaenichthys charcoti*; Pfla: *Perca flavescens*; Pflu: *Perca fluviatilis*; Sluc: *Sander lucioperca*; Ssin: *Sillago sinica*

A total of 38,490 gene models were predicted for *E. tetradactylum*, including 37,683 protein-coding genes and 807 tRNAs. Orthologous genes of the four-finger threadfin were compared to the genomes of goldfish, salmon, zebrafish and human. A total of 8777 gene groups are shared in these five vertebrates (Fig. 1a), while 12,071 gene groups are conserved among the four fish species. In addition, we carried out an orthologous gene comparison between the four-finger threadfin and all other available percid fish genomes (Fig. 1b). The number of protein coding genes of these 12 percid fish species ranges considerably, from 20,541 to 37,683 (Supplementary Table S2).

### Repeat content

To assess the repeat content of the four-finger threadfin genome, we generated a de novo repeat library using RepeatModeler. Following this, we applied two approaches for repeat annotation: (i) a standard RepeatMasker analysis, and (ii) a custom implementation including repeat defragmentation and removal of overlapping annotations. The standard RepeatMasker analysis identified a very low repeat content of just 9.07% (Table 2), while the custom implementation identified a slightly higher, but still very low repeat content of 10.91%, with the slight increase a consequence of merging fragmented repeats into longer repeat models during this approach (Table 2).

**Table 2:** Table summarising repeat content in the *E. tetradactylum* genome, detailing the number of elements, overall length, and genomic proportion, for each major repeat type, for a standard RepeatMasker analysis (left) and a custom annotation approach (right, see methods for details)

Repeat Class	Traditional Repeat Annotation (RepeatMasker)			Refined Conservative Repeat Annotation		
	No. elements	Total Length (Mb)	Percentage sequence (%)	No. elements	Total Length (Mb)	Percentage sequence (%)
**Retroelement**	155419	19.14	3.14	150453	21.92	3.59
*SINE*	13534	1.46	0.24	12936	1.63	0.27
*LINE*	60949	11.49	1.88	60294	12.18	1.99
*LTR element*	80936	6.19	1.01	77223	8.11	1.33
**DNA transposon**	298755	26.04	4.27	276340	32.42	5.31
**Rolling-circle**	15702	1.28	0.21	15557	2.05	0.34
**Unclassified**	32821	7.88	1.29	29890	7.80	1.28
**Other**	11396	1.00	0.16	19703	2.40	0.39
**Total repeats**	514093	55.34	9.07	491943	66.59	10.91

**Fig. 2:**
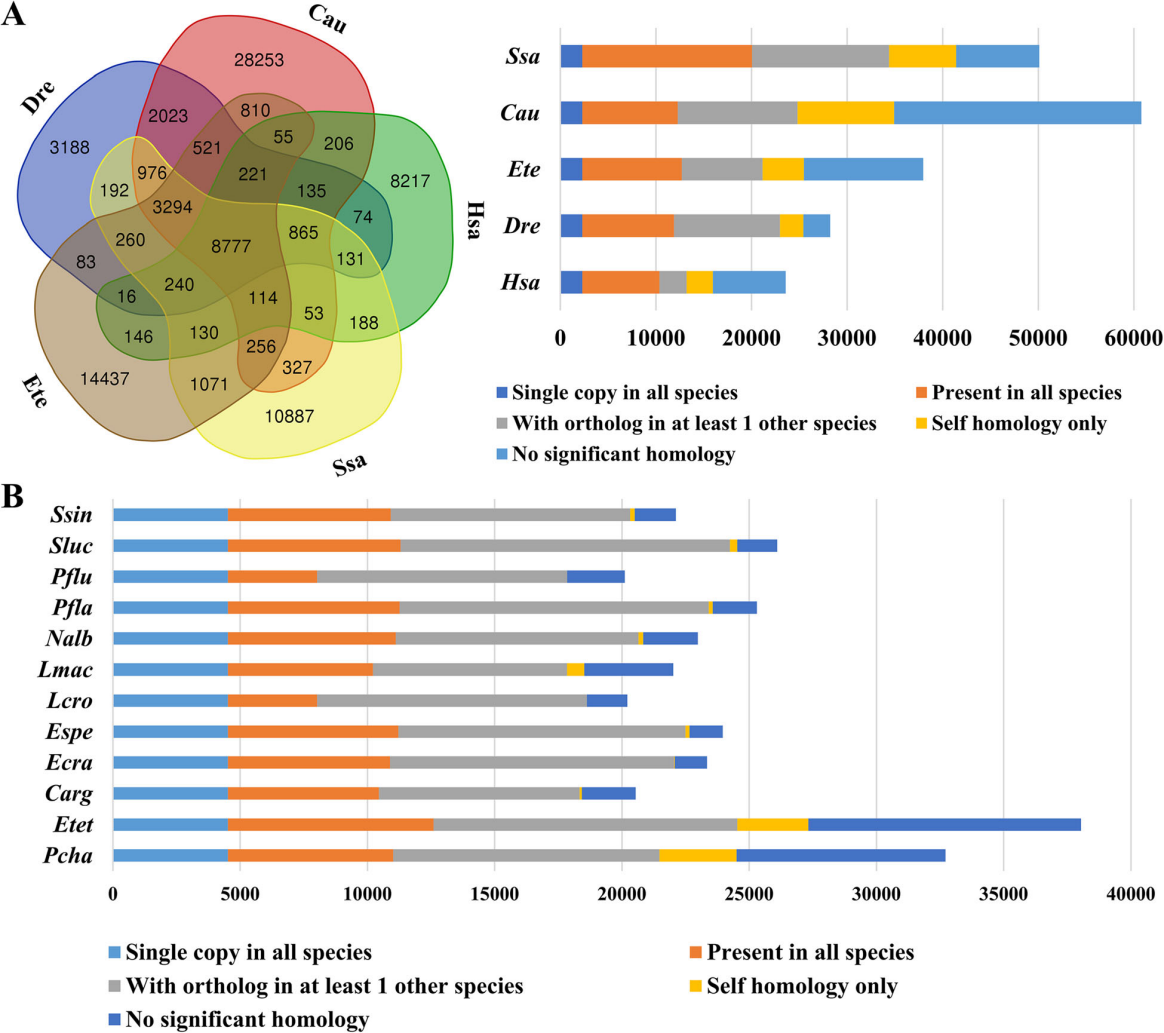
Summary of the repeat content of *E. tetradactylum*, showing (clockwise from top left): A photograph of the four-finger threadfin, a bar chart of the locations in which different repeat types are found within the genome, a repeat landscape plot, illustrating divergence levels for specific repeat types against the proportion of the genome for each level specified, and a pie chart of the proportion of major repeat types present in the genome. The image of the adult four-finger threadfin is modified from Wikipedia (user: BEDO, license: CC BY-SA 4.0, https://creativecommons.org/licenses/by-sa/4.0/), by removing the image background

In the *E. tetradactylum* genome, repeats are much more prevalent in gene flanks (regions within 20 kb upstream and 20 kb downstream of annotated host genes), compared to intergenic regions or introns (Fig. 2: Repeat Localities). This potentially suggests recruitment of repeats for host associated purposes through donation of coding or regulatory sequence (e.g. [9, 10]), although very little evidence exists of repeats directly inserted into gene regulatory 5′ and 3′ UTRs (Fig. 2: Repeat locality). Alternatively, this pattern suggests the involvement of other at present unclear genomic processes that have resulted in an uneven distribution of repeats.

Transposable elements (TEs) account for the vast majority of repeats annotated in the four-finger threadfin genome (Table 2). Other categories of repeat (simple, small RNA, satellite, and low complexity) account for just 0.32–1.01% of the genome (Table 2, Fig. 2: Repeat content). Among TEs, the largest contribution of sequence comes from DNA transposons (4.27–5.31%), followed by LINEs (1.88–1.99%%), and LTR elements (1.01–1.33%) (Table 2). These figures are broadly similar to those reported for the yellow drum *Nibea albiflora* [11], another perciform fish with a similarly low TE content.

Examination of the repeat landscape generated for the four-finger threadfin implies that there has been a steady decrease in transposon activity over recent time periods, since there is a notable decrease in repeats separated by low levels of divergence, which correspond to more recent copies (Fig. 2: Repeat landscape plot). This pattern is primarily a consequence of a reduction in the relative activity of DNA transposons and LTR elements, since levels of divergence for LINEs appears to have remained relatively stable.

### Hox, ParaHox, and sox genes of *E. tetradactylum*

**Fig. 3:**
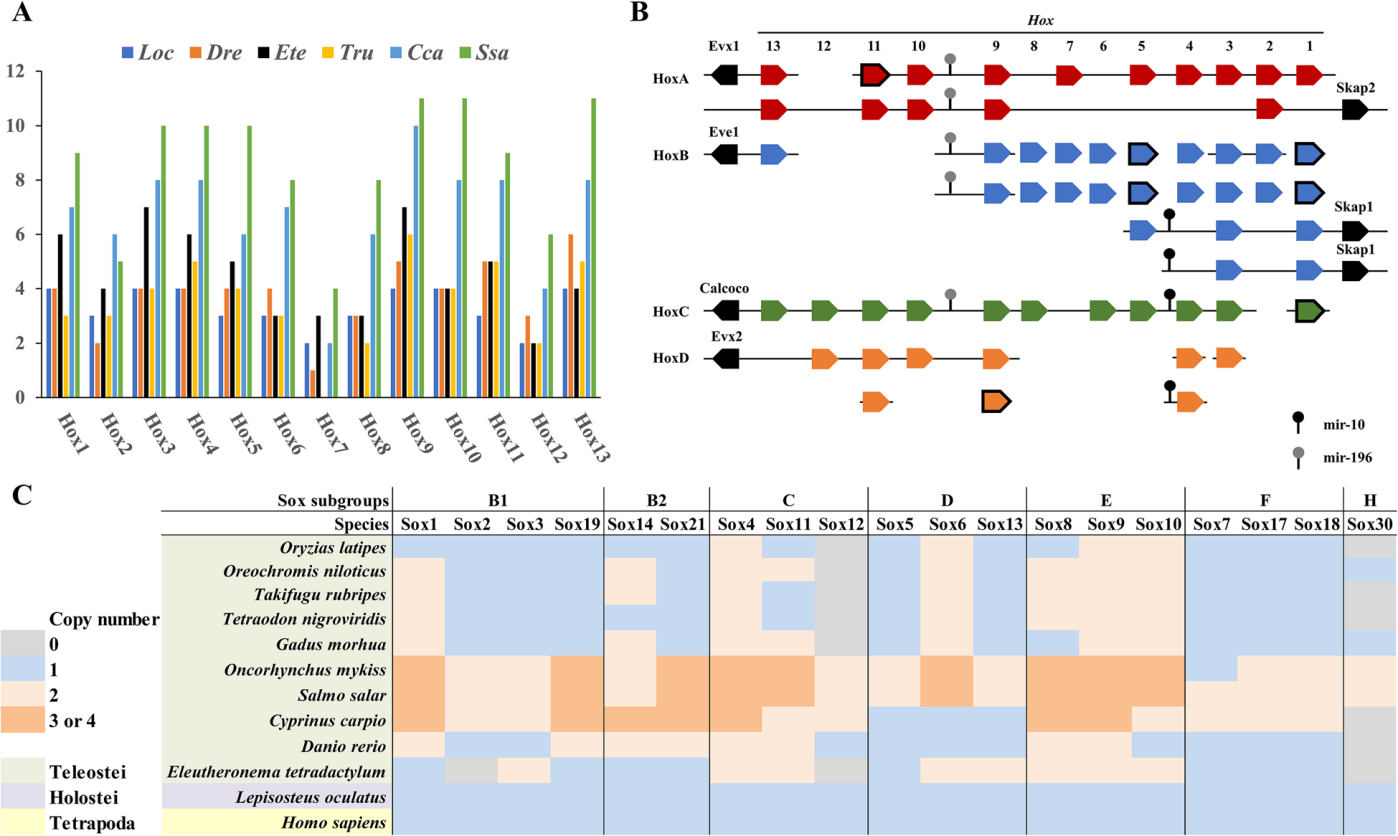
*Hox* and *Sox* genes of *E. tetradactylum*. **a** Hox gene numbers in different fishes; **b** Genomic organisation of *E. teradactylum Hox* genes. Bold box indicates only homeodomain being identified. **c** Comparison of *Sox* family genes between *E. tetradactylum* and other vertebrates. Abbreviations: Loc: spotted gar *Lepisosteus oculatus*, Dre: zebrafish *Danio rerio*, Ete: four-finger threadfin *Eleutheronema tetradactylum*; Tru: fugu *Takifugu rubripes*, Cca: common carp *Cyprinus carpio*, Ssa: Atlantic salmon *Salmo salar*

*Hox* cluster genes encode a group of transcription factors that control the anteroposterior axis during development [12, 13]. A total of 59 *Hox* genes were recovered in the *E. tetradactylum* genome (Fig. 3a-b, Supplementary data S1, Figure S2). HoxA, B, C, D clusters with conserved microRNAs (*mir-10* and *mir-196*) were revealed (Fig. 3b). Retention of *HoxA7*, extra copies of *HoxB* genes, as well as the loss of *HoxD13a* were observed in *E. tetradactylum* (Fig. 3b). These data suggest dynamic *Hox* gene gains and losses have occurred during fish evolution.

As for the *Hox* evolutionary sister group, the *ParaHox* genes including 2 *Gsx*, 1 *Pdx*, and 3 *Cdx* genes could be identified on 6 different scaffolds (Supplementary data S1, Figure S3). This situation mirrors what has been found in other teleosts, which have broken *ParaHox* clusters with secondary gene losses having occurred after whole genome duplication [14, 15].

The expansion of key transcription factors is proposed to act as a critical genetic driver for the evolution of vertebrate innovations [16]. The *Sox* gene family encodes transcription factor members that contain the high mobility group box (HMG box) DNA binding domain, which is conserved throughout the metazoans, and play vital roles in various developmental processes [17, 18]. A total of 26 *Sox* genes from 6 subgroups were identified in *E. tetradactylum* genome, but not *Sox30*, which is similar to findings for the zebrafish and pufferfish (Fig. 3c, Supplementary Figure S4).

### MicroRNAs

**Fig. 4:**
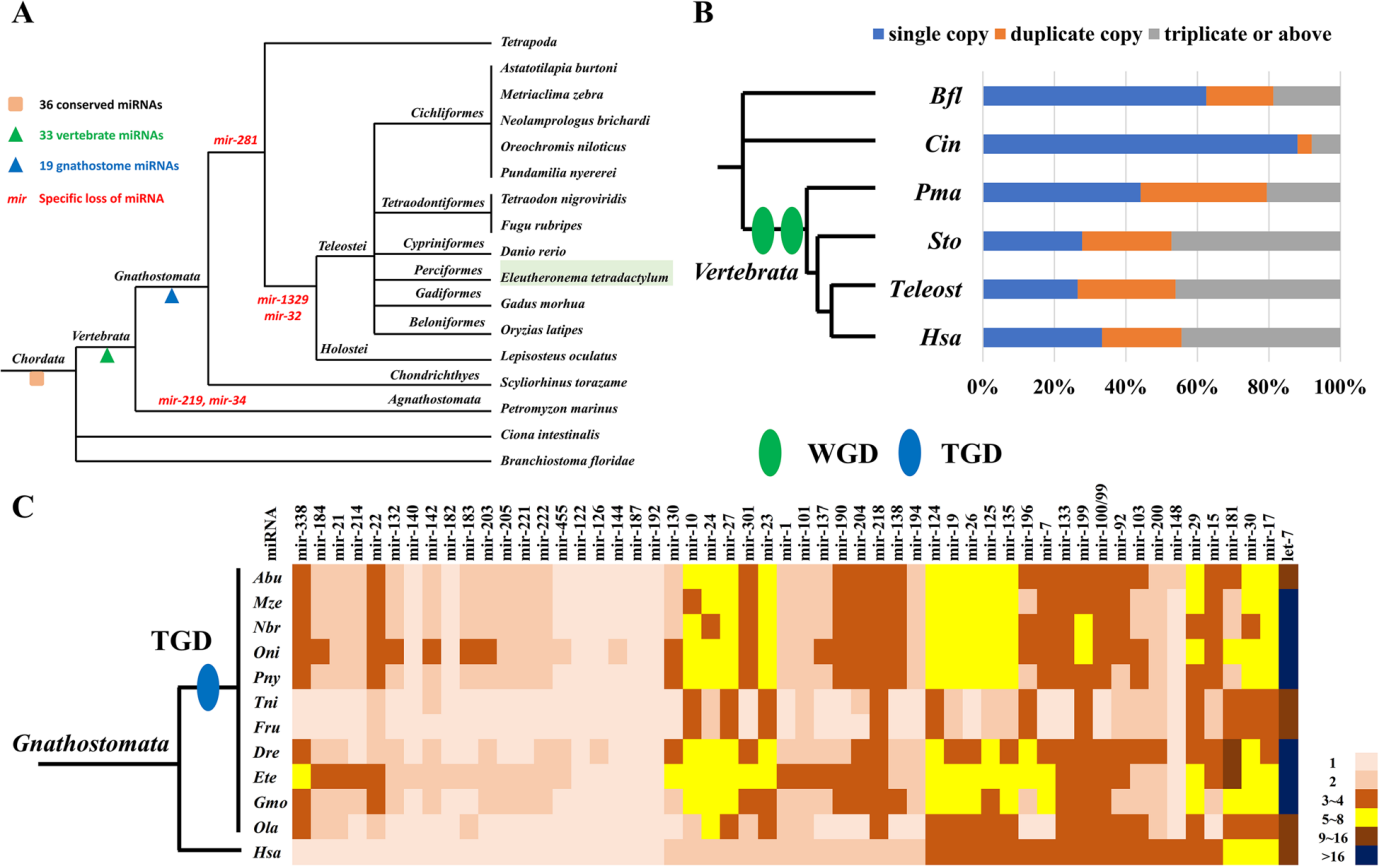
Evolution of chordate microRNAs. **a** Gains and losses of microRNAs in chordates; **b** Copy numbers of 36 chordate-conserved microRNAs; **c** Copy numbers of microRNAs in human and teleosts after teleost-specific genome duplication (TGD). Abbreviations: Bfl: *Branchiostoma floridae*, Cin: *Ciona intestinalis*, Pma: *Petromyzon marinus*, Sto: *Scyliorhinus torazame*, Hsa: *Homo sapiens*, Abu: *Astatotilapia burtoni*, Mze: *Metriaclima zebra*, Nbr: *Neolamprologus brichardi*, Oni: *Oreochromis niloticus*, Pny: *Pundamilia nyererei*, Tni: *Tetraodon nigroviridis*, Fru: *Takifugu rubripes*, Gmo: *Gadus morhua*, Ola: *Oryzias latipes*

MicroRNAs are an important group of post-transcriptional regulators with a key role in development. A total of 356 microRNA genes, including 126 bilaterian-conserved microRNA species, were identified in *E. tetradactylum* (Supplementary data S2). Comparing *E. tetradactylum* microRNA content with that of other chordates available in miRBase [19], MirGeneDB [20] and other relevant genomes [21], 36 chordate-conserved microRNAs were identified (Fig. 4a). In particular, 33 and 19 microRNAs appear to have emerged in the vertebrate ancestor and gnathostome ancestor respectively (Fig. 4a, Supplementary data S2).

The last common ancestor of vertebrates underwent two rounds of whole genome duplication (WGD), with an additional round of WGD that occurred in the ancestor of teleosts (i.e. the teleost specific genome duplication or ‘TGD’) [22–29]. In the salmonid lineage and the lineage containing the last common ancestor of carp and goldfish, an additional fourth WGD (4R) occurred [30, 31]. Consequently, copy numbers of the 36 chordate-conserved microRNAs discussed above were investigated, to investigate patterns in the retention of duplicates (Fig. 4b). About 60% of microRNAs have multiple copies in different vertebrates (post-WGD). While in more distant vertebrate relatives the pattern appears to differ, for example in the sea squirt and amphioxus, 88 and 62.5% of their microRNAs exist as single copies respectively (Fig. 4b). This is likely to be influence of WGDs in vertebrate lineages. Intriguingly, comparison of microRNA copies between human and teleosts (which were subject to the TGD), also revealed that more than 50% of microRNAs have more paralogues in teleost species than in human (Fig. 4c). For example, 36 out of 54 (67%) conserved microRNAs possess additional duplicates in *E. tetradactylum* compared to human (Fig. 4c). Our findings suggest that WGD events have a profound influence on the evolution of microRNA complements, and thus that microRNA landscapes may be a useful indicator of WGD events.

## Discussion

Considerable variation exists among the genome sizes of perciform fish, with the genome size of the threadfin lying very much at the lower end. At the upper end of the spectrum for perciform fish, the pikeperch (Percidae: *Sander lucioperca*) has an estimated genome size of 1014 Mb, and an assembly genome size of ~ 900 Mb [32], and the red sea bream (Sparidae: *Pagrus major*) has an estimated genome size of ~ 806 Mb, and an assembly genome size of 829.3 Mb. Meanwhile, at the lower end of the spectrum, the yellow drum (Sciaenidae: *Nibea albiflora*) has an estimated genome of 573–581 Mb and an assembly genome size of 596 Mb [11], and the Chinese sillago (Sillaginidae: *Sillago sinica*) has an estimated genome size of ~ 524 Mb, and an assembly genome size of just 534 Mb [33]. Comparison of various percid fish genomes showed that *E. tetradactylum* possesses the largest number of protein-coding genes, but the smallest average protein size, which may contribute to its small overall genome size, despite its large number of genes.

Nguinkal et al. [32] examined repeat content among the genomes of nine perciform fish species, and found it to vary from 13.8% in the yellow drum (*Nibea albiflora*) to 39.8% in the pikeperch (*Sander lucioperca*). Thus, among perciform fish, the four-finger threadfin has an especially low repeat content. One of the key determinants of genome size in eukaryotes is repeat content [34], and in line with this, a strong relationship between genome size and repeat content is reported for perciform fish [32]. Consequently, the repeat content of the four-finger threadfin conforms to its relatively small genome size of ~ 610 Mb. However, the genome assembly of the yellow drum is even smaller (~ 565 Mb) than the four-finger threadfin genome presented here, but its repeat content is higher at 13.8% [11]. Thus, it appears that the repeat content of the four-finger threadfin is especially low, both among fish genomes [35], and compared to other vertebrate genomes more generally. Reasons for the low repeat content of the threadfin genome are unclear at present, and further elucidation of this finding represents an interesting avenue for future study. Further, it remains unclear what factors have led to certain expansions of repeats, particularly DNA transposable elements, in other perciform fish such as the pikeperch [32].

Hox cluster genes encode a group of transcription factors that control the anteroposterior axis during development [12, 13]. The *E. tetradactylum* contains *HoxA7*, while cyprinid fish (such as zebrafish, common carp and goldfish) and pufferfish have lost *HoxA7* [36–38]. Moreover, additional copies of *HoxB* genes, as well as the loss of *HoxD13a*, were observed in *E. tetradactylum*. These data suggest dynamic *Hox* gene gains and losses have occurred during fish evolution, and have undoubtedly helped to shape the wide diversity of body forms observable among teleost fish, and not least structures such as the charismatic threadfins displayed by fish in the family Polynemidae.

Different to majority of invertebrates, vertebrate ancestor has gone through 2R WGD and teleost ancestor has further experienced an extra TGD, which contributed to the greatly successful radiation and diversification of their genetic complexity [22–29]. By comparing microRNA contents in different chordate lineages (Fig. 4), the emergence/gain of microRNAs well reflected the functional consequences of vertebrate WGD, and the paralogue numbers of conserved microRNAs in various chordate lineages are also in line with those WGD events. Our findings suggest that microRNA landscape could be a useful indicator of WGD event.

## Conclusions

This study provides a new genomic resource of the four-finger threadfin *E. tetradactylum*, and represents the first available genome sequence for the biologically interesting and economically important threadfin fishes (family Polynemidae)*.* In particular, our analyses help to facilitate studies on the developmental biology of the four-finger threadfin, and comparative developmental genomics analyses among perciform fish more generally. With their great diversity, wide variation in body forms, and specialized morphological adaptations, in combination with rapidly increasing genomic resources, the perciform fishes are fast emerging as a vertebrate group with great potential to further the study of developmental genomics. Additionally, we provide a detailed analysis of the repeat content of the four-finger threadfin genome, highlighting the very low repeat content present. It is currently unclear why repeat content varies so greatly among perciform fish, and what mechanisms drive largescale reductions in repeat content in certain genomes. However, the analysis of unsampled lineages from across phylogenetic diversity, as undertaken here, lays important groundwork for further exploration and elucidation of these patterns. Overall, the analyses and genomic resources provided here provide a starting point for further advances in our understanding of the genomics of the unusual threadfin fishes.

## Methods

### Genomic DNA extraction, sequencing and assembly

Frozen flesh of a single individual of *E. tetradactylum* was obtained from a Sai Kung market at Sai Kung, Hong Kong. Genomic DNA was isolated from muscle tissue using the PureLink Genomic DNA Kit (Invitrogen), and species identity was confirmed with *COI* barcoding. The DNA sample was sent to Novogene (Hong Kong) for library preparation and sequencing on the Illumina HiSeq X system. Chromium WGS reads were assembled using Supernova (v2.1.1) with default parameters (https://support.10xgenomics.com/de-novo-assembly/software/pipelines/latest/using/running), and the Supernova pseudohap assembly output was used for further analysis. Genome size estimation was analyzed using a k-mer-based statistical approach in the GenomeScope webtool [39]. Completeness of genome assembly was examined by BUSCO (v4.0.0, metazoa_odb10, actinopterygii_odb10, 8).

### Repetitive elements annotation

Repetitive elements were identified as previously described pipeline [40, 41] with the *chordata* RepBase dataset [42]. Subsequently, the resulting de novo repeat library was utilised to identify repetitive elements using RepeatMasker [43], by implementing two approaches. Firstly, a standard RepeatMasker analysis was performed. Secondly, repeat models were maximised using an automated process implemented in RepeatCraft [44] under strict merge parameters with LTR_FINDER v1.0.5 [45] and the LTR_FINDER_Parallel wrapper [46] to defragment repeat segments. For loci where RepeatMasker annotations overlapped (i.e where the same sequence was annotated as different repeat families), only the longest repeat was selected. This is a conservative approach that ensures TE content estimates are not inflated by counting the same bases multiple times, and facilitates a one-to-one matching of sequence with repeat family identity. A revised summary table was constructed using the revised repeat counts for the second approach, which are presented alongside the bare RepeatMasker results. Rstudio v1.2.1335 [47] with R v3.5.1 [48] and ggplot2 ver. 3.2.1 [49] was used to generate all the plots.

### Genome and microRNA annotation

Raw sequencing reads from 4 transcriptome datasets were downloaded from the Sequence Read Archive (SRA) (SRR7899951, SRR7899952, SRR7899953 and SRR7899954) for gene model prediction using Trimmomatic [50], Funannotate [51], Trinity [52] and PASA [53] as previously described procedures and parameters [41, 54].

Precursor sequences of microRNAs of known chordate species were retrieved from both miRbase and MirGeneDB (Supplementary data S3, [19, 20]), and used to search for homologous sequences in the *E. tetradactylum* genome using BLASTN with the following parameters: -r 5 -q − 4 -G 8 -E 6 -e value 1. Results were also manually inspected for good sequence conservation and hairpin folding by CentroidFold [55].

### Gene family and phylogenetic analyses

Gene models of goldfish (*Carassius auratus*), zebrafish (*Danio rerio*), salmon (*Salmo salar*) and human (*Homo sapiens*) were download from Goldfish genome project (https://research.nhgri.nih.gov/goldfish) and NCBI (GCF_000002035.6, GCF_000233375.1, GCF_000001405.39) respectively, and further compared with *E. tetradactylum* using all-against-all BLASTP alignment (*E*-value of 10^− 5^) and OrthoMCL (v2.0.9, inflation value of 1.5, [56]). The links to the gene models of 12 percid fish are shown in Supplementary Table S3. The gene models were then compared with *E. tetradactylum* using the same parameters.

For analysis of *Hox*, *ParaHox*, and *Sox* genes, reference sequences were obtained from HomeoDB ([57], http,//homeodb.zoo.ox.ac.uk/), NCBI (https://www.ncbi.nlm.nih.gov/), Uniprot (https://www.uniprot.org/) and relevant genomes in Ensembl database (https://asia.ensembl.org/index.html) as queries to carry out tBLASTn [58] searches to retrieve protein coding gene sequences from the *E. tetradactylum* genome (reference sequences used in this study could be found in Supplementary data S3). Each putatively identified gene was also compared to sequences in the NCBI nr database. Further, protein sequences were aligned to other known members of putative gene families using MAFFT [59], and phylogenetic trees were constructed using MEGA [60] and displayed using iTOL [61].

## Supplementary information

**Additional file 1.** (XLSX 49 kb)

**Additional file 2: Table S1.** Results of BUSCO assessment. **Table S2.** Protein-coding genes in various percid fish. **Table S3.** Details of the genome, protein, and gff files of species used in this study. **Figure S1.** Genome size estimation of *E. tetradactylum*. **Figure S2**. Phylogenetic analysis of Hox genes by Neighbor-Joining (A) and Maximum Likehood (B) method. **Figure S3.** Phylogenetic analysis of ParaHox genes by Neighbor-Joining (A) and Maximum Likehood (B) method. **Figure S4.** Phylogenetic analysis of Sox family genes by Maximum Likehood (A) and Neighbor-Joining (B) method. (XLSX 49 kb)

## Data Availability

The genome assembly data can be found at NCBI with accession number WFKG00000000. Datasets of SRR7899951, SRR7899952, SRR7899953, SRR7899954 were retrieved from NCBI (https://www.ncbi.nlm.nih.gov/). Gene models of goldfish (*Carassius auratus*), zebrafish (*Danio rerio*), salmon (*Salmo salar*) and human (*Homo sapiens*) were downloaded from Goldfish genome project (https://research.nhgri.nih.gov/goldfish) and NCBI (https://www.ncbi.nlm.nih.gov/, GCF_000002035.6, GCF_000233375.1, GCF_000001405.39). The sources for obtaining gene models of 12 percid fish are shown in Supplementary Table S3, and the microRNA precursor sequences of known chordate species and reference sequences of *Hox*, *ParaHox* and *Sox* genes are shown in Supplementary data S3.

## Abbreviations

WGD: Whole genome duplication
TGD: Teleost specific genome duplication
WGS: Whole genome sequencing
Cau: Goldfish *Carassius auratus*
Dre: Zebrafish *Danio rerio*
Ete/ Etet: Four-finger threadfin *Eleutheronema tetradactylum*
Hsa: Human *Homo sapiens*
Ssa: Atlantic salmon *Salmo salar*
Carg: *Channa argus*
Ecra: *Etheostoma cragini*
Espe: *Etheostoma spectabile*
Lcro: *Larimichthys crocea*
Lmac: *Lateolabrax maculate*
Nalb: *Nibea albiflora*
Pcha: *Parachaenichthys charcoti*
Pfla: *Perca flavescens*
Pflu: *Perca fluviatilis*
Sluc: *Sander lucioperca*
Ssin: *Sillago sinica*
Loc: spotted gar *Lepisosteus oculatus*
Tru/ Fru: Fugu *Takifugu rubripes*
Cca: Common carp *Cyprinus carpio*
Bfl: *Branchiostoma floridae*
Cin: *Ciona intestinalis*
Pma: *Petromyzon marinus*
Sto: *Scyliorhinus torazame*
Abu: *Astatotilapia burtoni*
Mze: *Metriaclima zebra*
Nbr: *Neolamprologus brichardi*
Oni: *Oreochromis niloticus*
Pny: *Pundamilia nyererei*
Tni: *Tetraodon nigroviridis*
Gmo: *Gadus morhua*
Ola: *Oryzias latipes*
