# Supplementary material for: Genome of the four-finger threadfin Eleutheronema tetradactylum (Perciforms: Polynemidae)
Source: BMC Genomics. 2020 Oct 19;21:726. doi: 10.1186/s12864-020-07145-1 (PMC7574432; doi:10.1186/s12864-020-07145-1)

**Table S1. Results of BUSCO assessment.**

| **Lineage dataset** | **metazoan_odb10** | **actinopterygii_odb10** |
| --- | --- | --- |
| Complete BUSCOs (C) | 841 (88.2%) | 3069 (84.3%) |
| Complete and single-copy BUSCOs (S) | 739 (77.5%) | 2749 (75.5%) |
| Complete and duplicated BUSCOs (D) | 102 (10.7%) | 320 (8.8%) |
| Fragmented BUSCOs (F) | 79 (8.3%) | 236 (6.5%) |
| Missing BUSCOs (M) | 34 (3.5%) | 335 (9.2%) |
| Total BUSCO groups searched | 954 | 3640 |

**Table S2. Protein-coding genes in various percid fish.**

**Table S3. Details of the genome, protein, and gff files of species used in this study.**

**Figure S1. Genome size estimation of *E. tetradactylum*.**


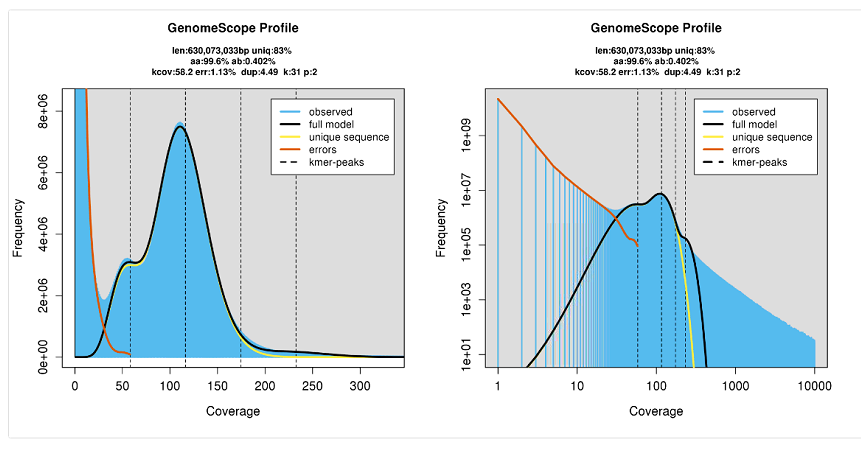


**Figure S2. Phylogenetic analysis of Hox genes by Neighbor-Joining (A) and Maximum Likehood (B) method.** The trees are drawn to scale and inferred from 1000 bootstraps. The bootstrap value is indicated by color range.

**A)**


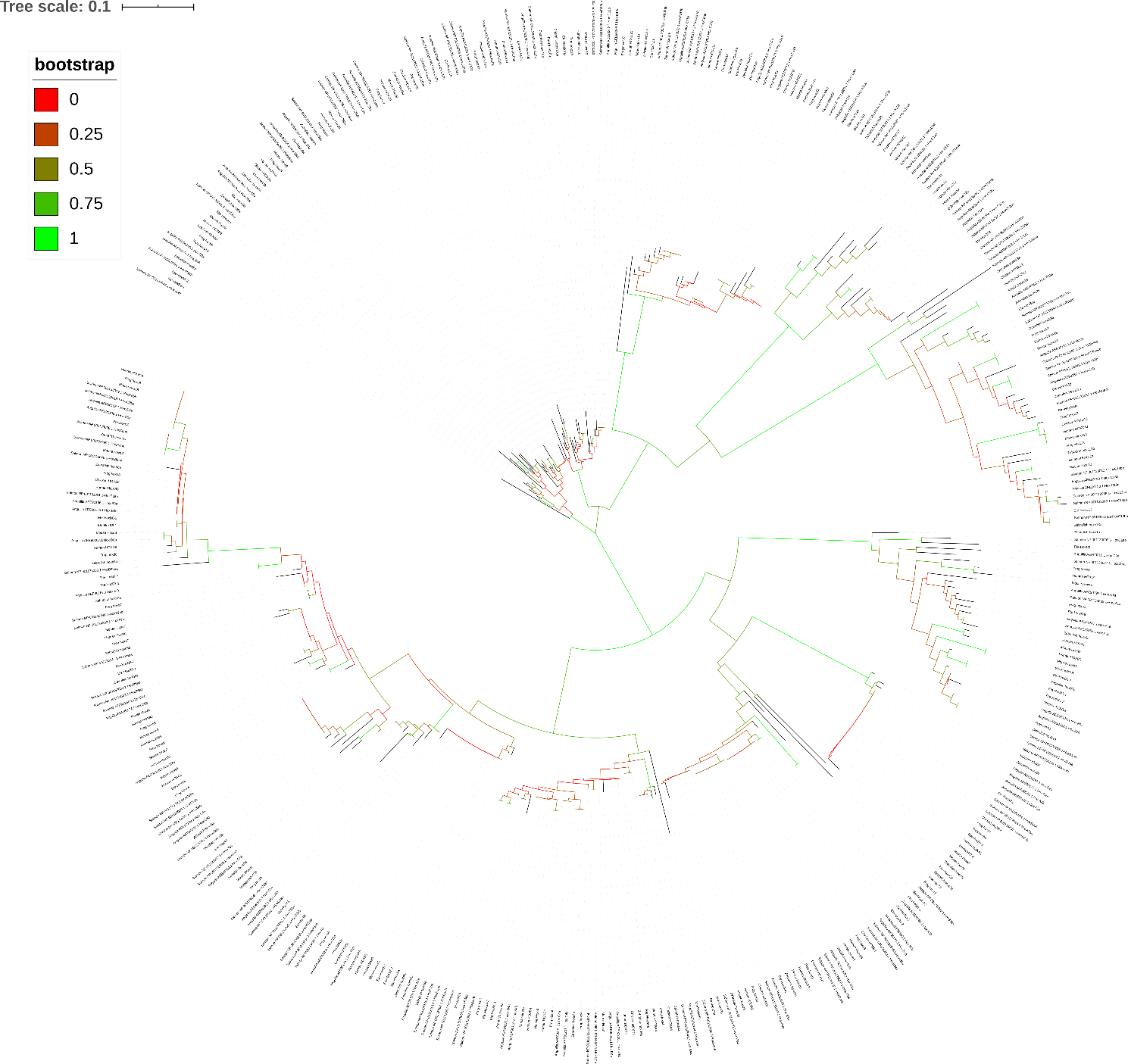


**B)**


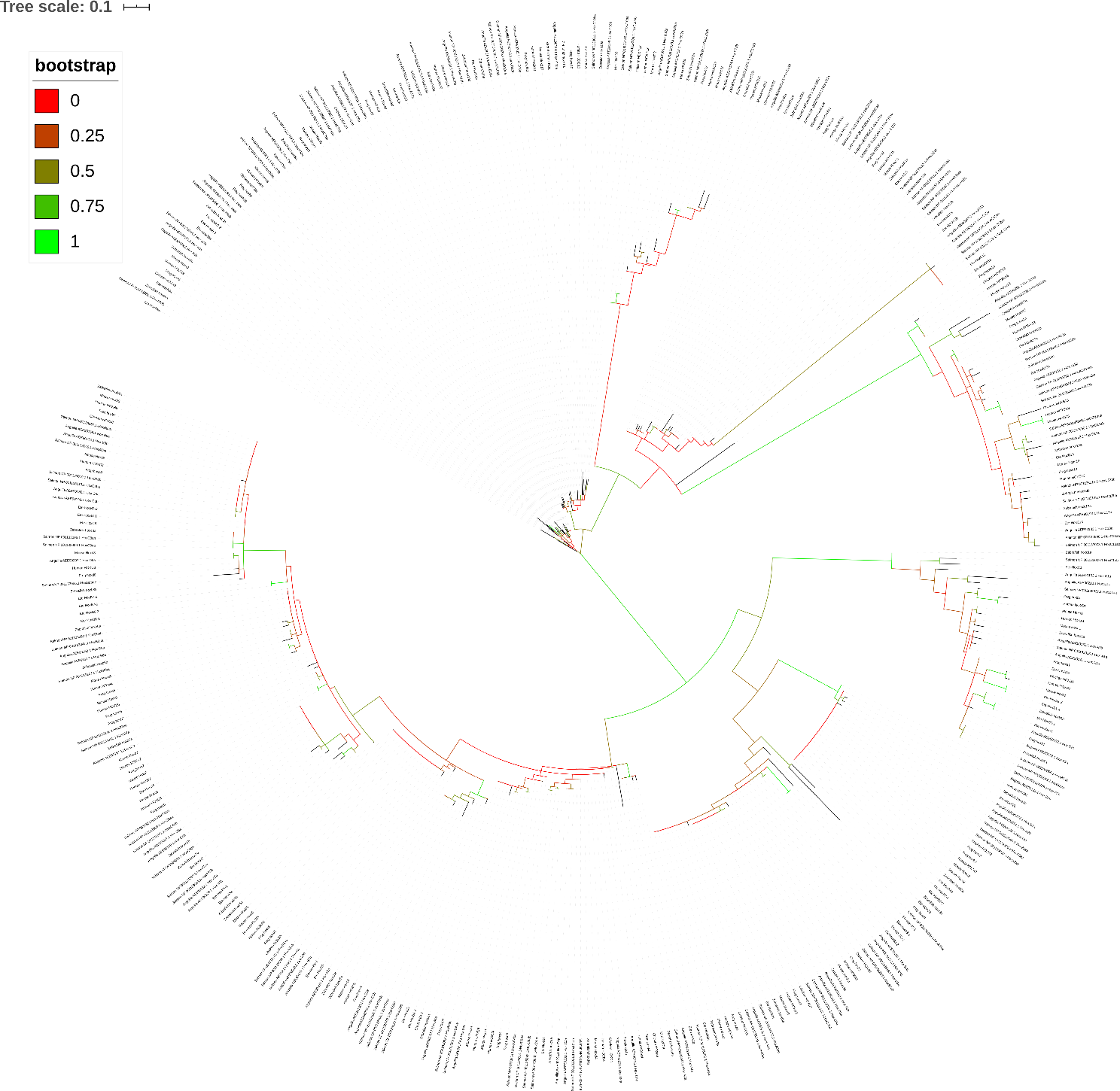


**Figure S3. Phylogenetic analysis of ParaHox genes by Neighbor-Joining (A) and Maximum Likehood (B) method.** The trees are drawn to scale and inferred from 1000 bootstraps. ParaHox genes of *E. tetradactylum* are highlighted in green dot.

**A)**

**B)**

**Figure S4. Phylogenetic analysis of Sox family genes by Maximum Likehood (A) and Neighbor-Joining (B) method.** The tree is drawn to scale and inferred from 1000 bootstraps. The bootstrap value is indicated by color range.

**A)**


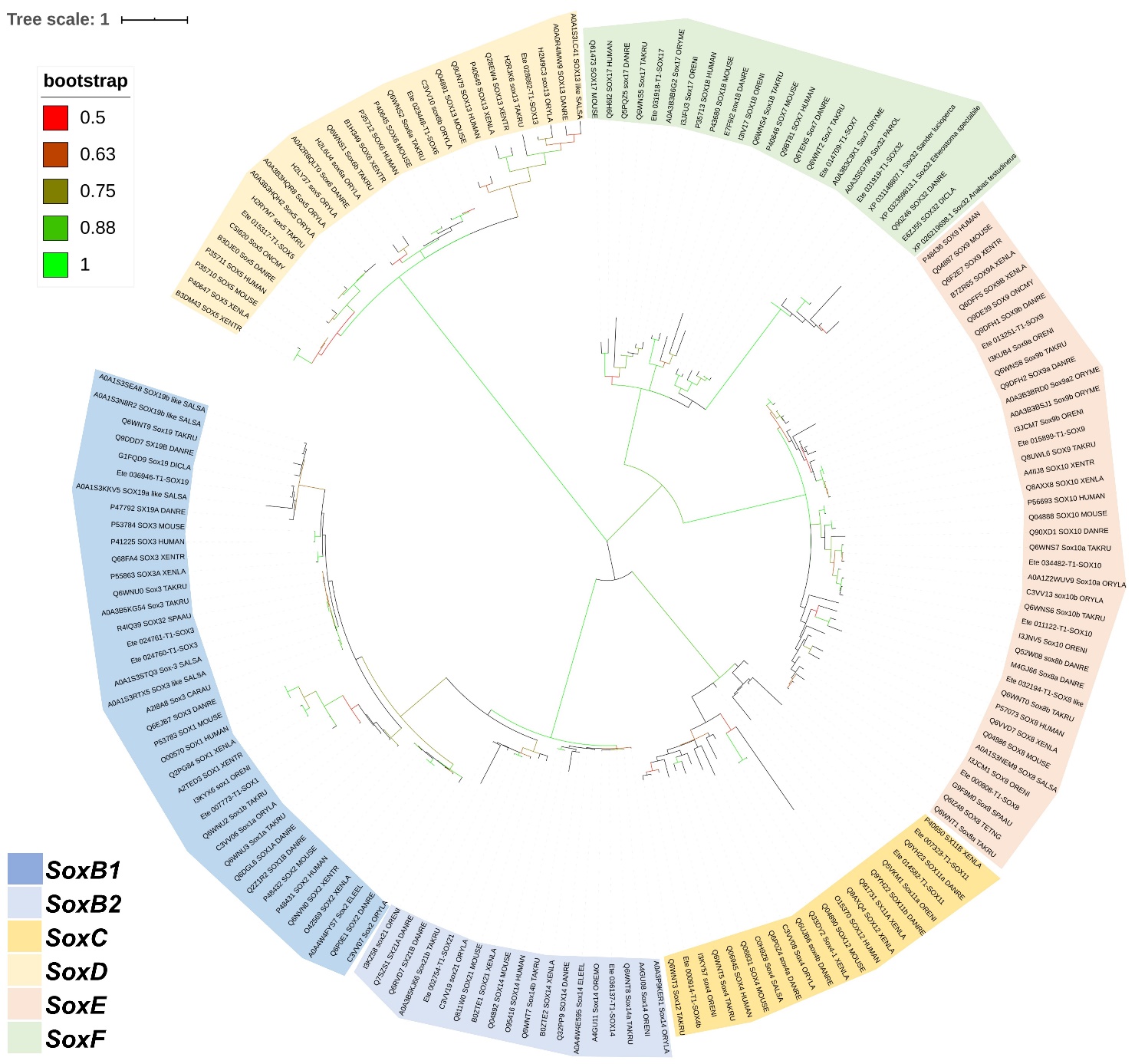


**B)**


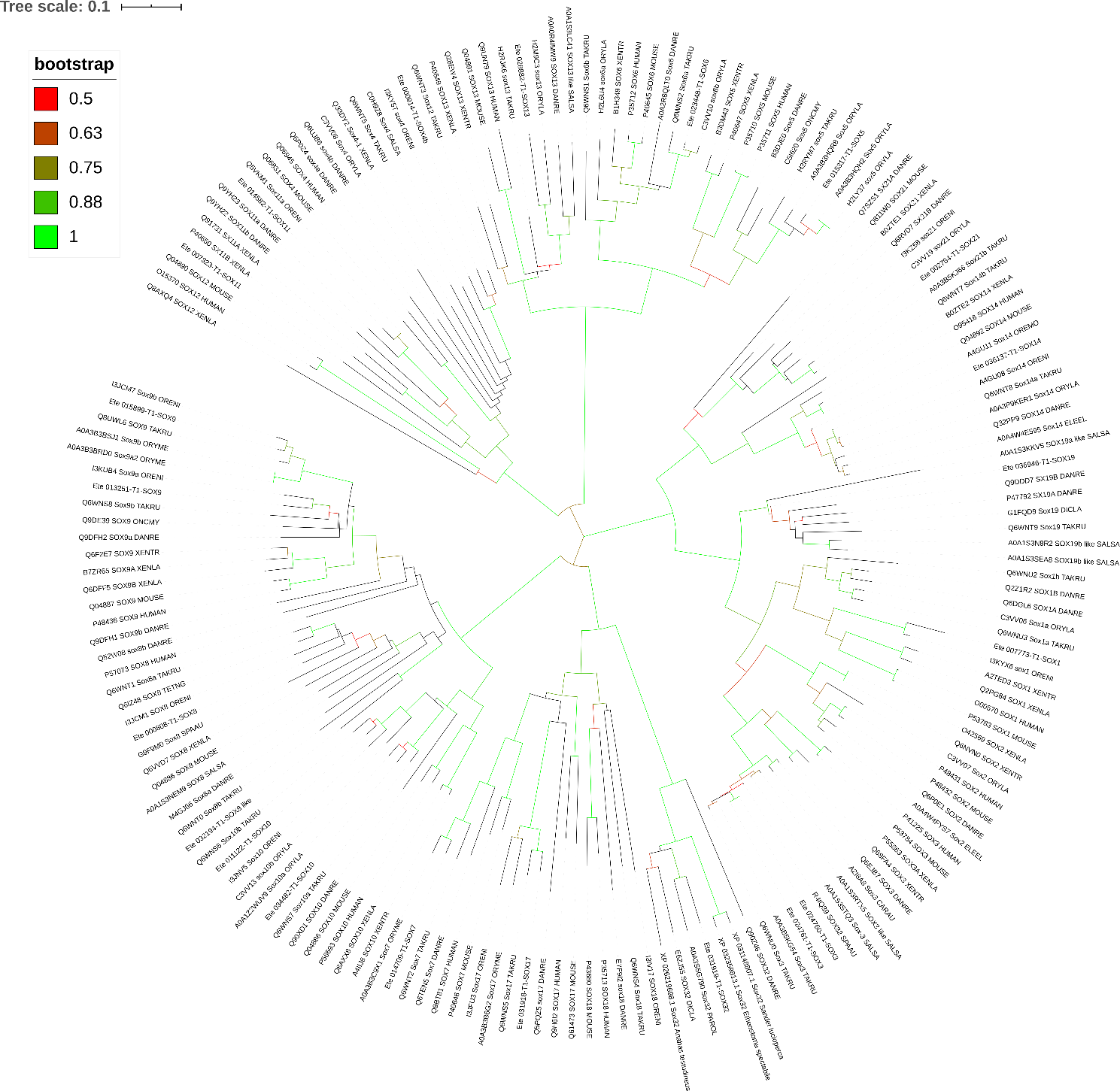

Supplement: Supplementary file 2 — Additional file 2: Table S1. Results of BUSCO assessment. Table S2. Protein-coding genes in various percid fish. Table S3. Details of the genome, protein, and gff files of species used in this study. Figure S1. Genome size estimation of E. tetradactylum. Figure S2. Phylogenetic analysis of Hox genes by Neighbor-Joining (A) and Maximum Likehood (B) method. Figure S3. Phylogenetic analysis of ParaHox genes by Neighbor-Joining (A) and Maximum Likehood (B) method. Figure S4. Phylogenetic analysis of Sox family genes by Maximum Likehood (A) and Neighbor-Joining (B) method. (XLSX 49 kb) [file 12864_2020_7145_MOESM2_ESM.docx]
